# Editorial: ADHD: Science and society

**DOI:** 10.3389/fpsyt.2022.1129728

**Published:** 2023-01-16

**Authors:** Laura Batstra, Martin Whitely, Sami Timimi

**Affiliations:** ^1^Department of Child and Family Welfare, University of Groningen, Groningen, Netherlands; ^2^John Curtin Institute of Public Policy, Curtin University, Perth, WA, Australia; ^3^Child and Adolescent Mental Health Service, Lincolnshire Partnership NHS Foundation Trust, Lincoln, United Kingdom

**Keywords:** ADHD, reification, mental disorders, medicalization, DSM

Since 1980, when Attention Deficit/Hyperactivity Disorder (ADHD) replaced Hyperkinetic Disorder in the third edition of the Diagnostic and Statistical Manual of Mental Disorders (1), there has been an extensive search for brain related and genetic biomarkers. Despite numerous promises of imminent breakthroughs, decades later we are no nearer to knowing anything tangible about the hypothesized biological underpinnings of ADHD.

This failure raises important questions that require new modes of enquiry. What role might psychosocial circumstances play? How does the belief that ADHD is a disease effect institutional practice? What unintended consequences for individuals and societies might arise from popularizing the idea that these common behaviors are potential diseases? For this Research Topic “ADHD: Science and Society” we invited authors to probe beyond bio-deterministic models and explore these multi-layered questions.

The articles, draw attention to the impact of personal and professional interests, power issues, assumptions of the DSM, and to processes like reification, throwing light on how these have encouraged diagnosis and medication prescriptions.

Mills reveals how ADHD was accepted into the third edition of DSM as the result of careful political maneuvering, not scientific endeavor. In the 1970s psychiatry experienced a crisis of credibility and many insurance companies in the US questioned the lack of specificity in the methods used for assessment and treatment. DSM-III provided a way for standardizing practice that could be used for categorizing presentations and improving the efficiency of reimbursement bureaucracies. Despite these political and economic origins and numerous subsequent criticisms (2–4), the belief that ADHD was a discrete and knowable medical condition became dominant.

Koutsoklenis and Honkasilta point out how DSM-5-TR, published in 2022, explicitly and implicitly maintains the essentialist medical scientific metaphor of disorder. For example, it states that “prevalence is higher in special populations such as foster children or correctional settings,” but discussion about the impact of adverse life trajectories is missing. Other social factors like economic disadvantage, social status, and familial care, which Erlandsson et al. discuss in their applied mixed-method systematic review, are also not mentioned. Such textual silence, according to te Meerman et al., is one of the at least four mechanisms that reify the ADHD and portray it as a disease entity that explains rather than just describes inattentive and restless behaviors.

Ponnou and Thomé, using comprehensive French general health insurance scheme data, showed that French children and adolescents who are the youngest in their class, as well as children from disadvantaged families, are more likely to be diagnosed with ADHD and prescribed methylphenidate. This resonates with other international studies that have documented that the youngest children in a school year are considerably more likely to be diagnosed with and/or medicated for ADHD than their older classmates (5, 6). None of the DSM editions however, mention this relative age effect.

Besides such textual silence, other reifying mechanisms described by te Meerman et al., are language choice, logical fallacies, and genetic reductionism. Koutsoklenis and Honkasilta show that DSM-5 engages in genetic reductionism by stating that heritability of ADHD is 74%, without mentioning that this estimate stems from twin studies that cannot separate environmental from genetic influences (7). In addition, they point to a logical fallacy in DSM-5-TR when it retains the circular logic of previous editions, i.e., you know an individual has ADHD because they are inattentive, disorganized, and/or hyperactive-impulsive, but the reason they are inattentive, disorganized and/or hyperactive-impulsive is because they have ADHD.

To illustrate how language choice and metaphors can reify the abstract, te Meerman et al. refer to Russel Barkley, an influential ADHD advocate, who compares ADHD to a meat-cleaver: “*The back part of your brain is knowledge, the front part is performance. ADHD, like a meat cleaver, just split your brain in half.”* The meat-cleaver metaphor arguably has a strong “fear appeal” and reifies the construct of ADHD by removing the agency of individuals. But like all such “neuro” based theories, it lacks any empirical support. Mills deconstructs Barkley's flimsy logic, showing how rhetoric is used to obscure the lack of positive research findings, but that the subsequent dominance of brain-based research and language makes it seem “scientific.”

According to Schleim it is unlikely that biomarkers for ADHD and many other mental disorders will ever be established: the diagnostic categories are too broad for it to be plausible that DSM definitions neatly match people's behaviors and experiences on the one hand and specific brain anatomy/physiology on the other. Nevertheless, Erlandsson et al. expect that the vast majority of research on inattentive and hyperactive child behaviors will continue to rest on scientifically unjustifiable assumptions about children's attributes. The unequal allocation of resources available in contemporary ADHD research is worrisome, especially considering recent research indicating the potential negative effects of the ADHD label (8) and its treatment (9).

Collectively, the papers published in this Research Topic edition invite the question: If the science supporting ADHD diagnosis and treatments is weak and has not advanced significantly since 1980; What is driving increased ADHD diagnosis and treatment rates?

With the global ‘ADHD therapeutics market' estimated to be worth US$29.56 billion in 2022—and expected to reach US$45.68 billion by 2027 (10)—perhaps the answer lies in thinking differently about ADHD. Instead of regarding it as an illness requiring medical intervention, it may be more insightful to understand ADHD as a marketable brand driven by economic and guild interests.

An example of a possible catalyst of change is the set of guidelines on ADHD psycho-education, described by te Meerman et al. In addition, the underlying motives of stakeholders and their opposing interests need further investigation. Identifying and hopefully eliminating the impact of any misalignment between parties with a self-interest on the one hand, and the wellbeing of the consumers of ADHD on the other, should be a priority.

We hope our Research Topic will provide food for thought and a springboard for academics, scholars, and practitioners to expand their efforts in reimagining this condition in order to prevent further harm being unnecessarily visited on our populations.

## Author contributions

All authors listed have made a substantial, direct, and intellectual contribution to the work and approved it for publication.
